# Changes in the Selected Antioxidant Defense Parameters in the Blood of Patients after High Resolution Computed Tomography

**DOI:** 10.3390/ijerph16091476

**Published:** 2019-04-26

**Authors:** Amira Bryll, Wirginia Krzyściak, Anna Jurczak, Robert Chrzan, Anna Lizoń, Andrzej Urbanik

**Affiliations:** 1 Department of Radiology, Jagiellonian University Medical College, Kopernika 19, 31-501 Krakow, Poland; bryllamira@gmail.com (A.B.); robert.chrzan@uj.edu.pl (R.C.); andrzej.urbanik@uj.edu.pl (A.U.); 2 Department of Medical Diagnostics, Faculty of Pharmacy, Jagiellonian University Medical College, Medyczna 9, 30-688 Krakow, Poland; anna.lizon@uj.edu.pl; 3 Department of Pediatric Dentistry, Institute of Dentistry, Jagiellonian University Medical College, Montelupich 4, 31-155 Krakow, Poland; anna.jurczak@uj.edu.pl

**Keywords:** ionizing radiation, reactive oxygen species, human antioxidant systems

## Abstract

Ionizing radiation generated during high resolution computed tomography (HRCT) scanning may have an indirect effect on the mechanisms regulating the oxidative-antioxidant balance in the human body, which is one of the necessary factors ensuring the maintenance of its homeostasis. The aim of the study was to analyze the response of antioxidant systems through the determination of the antioxidant markers in the blood of patients exposed to oxidative stress resulting from the routine HRCT examination of the chest. Blood of 35 people aged 60.77 ± 10.81 taken before and at four time points after the examination constituted the test material. The determination of the total antioxidant capacity expressed as ferric reducing ability of plasma (FRAP) and ferric reducing antioxidant activity and ascorbic acid concentration (FRASC) were performed together with an examination of catalase activity and the concentration of the reduced glutathione. The organism’s response to ionizing radiation was associated with a significant decrease in the antioxidant markers’ levels at all time-points and showed a significant negative correlation depending on the radiation dose. Visible down-regulation of these markers is a response to increased oxidative stress. In light of the obtained results, the measurement of the selected markers of antioxidant defense may be a useful parameter of oxidative stress caused by ionizing radiation.

## 1. Introduction

Ionizing radiation during radiological examinations influences the initiation of free radical reactions in the body. After passing through the cell membrane, ionizing radiation causes the formation of oxygen free radicals by affecting water molecules. The water molecule undergoes radiolysis producing a hydrated electron (e^−aq^) and cation radical H_2_O^•+^, which undergoes rapid disintegration to a hydroxyl radical ^•^OH.

Homolytic cleavage of water and the formation of H^•^ and a hydroxyl radical is another possible route of reactive oxygen species formation:

H_2_O + hv → H_2_O^•^ → H^•^ + ^•^OH

This process ends in the formation of hydrogen and hydrogen peroxide molecules.

H^•^ + H^•^ → H_2_

^•^OH + ^•^OH → H_2_O_2_

Oxygen free radicals are therefore atoms, molecules or their fragments containing one or more unpaired electrons in an atom or molecular orbitals, and mainly determining much higher chemical reactivity as compared to their parental molecules.

The damaging effect of free radicals associated with their destructive effect on biological systems is described in the literature as an oxidative stress or nitrosative stress in the case of reactive nitrogen species (NOS). It occurs in cases of Reactive Oxygen Species/Nitric Oxides (ROS/NOS) overproduction with concomitant insufficiency or deficiency of enzymatic and non-enzymatic antioxidants.

Oxidative stress is therefore a result of an imbalance between pro-oxidative and antioxidative reactions with a shift towards the formation and accumulation of free radicals together with an occurrence of negative consequences in metabolism.

Ionizing radiation (IR) leads to the production of oxygen free radicals (ROS), which are a type of pro-oxidant that can cause indirect cell damage when their excessive production surpasses the detoxification capabilities of antioxidant systems. IR-induced ROS production has been proven in both in vitro and in vivo studies [1,2].

In addition to the hydroxyl radical (OH^•^), particle ionization results in the formation of other reactive oxygen species such as the superoxide anion radical (O_2_^•−^). On the other side, reactions of oxygen free radicals with proteins, lipids and nucleic acids lead to the formation of organic free radicals.

Such a mechanism may take place during radiological examinations as well as computed tomography (CT). This constitutes a significant problem because over the last twenty years, the use of CT in routine medical diagnostics has more than doubled and constitutes about 35% of all imaging tests [3,4].

Despite the high frequency of CT examinations, knowledge of the long-term effects of exposure to ionizing radiation in humans is still limited. Such an exposure may pose a potential threat to health and life of the human organism, including increased incidence of cancer [5].

The estimated risk associated with the development of cancer after exposure to ionizing radiation is usually extrapolated from animal models or in vitro tests without assessment of actual DNA damage. The results obtained from animal models clearly show that cellular response to damage (including oxidative stress) during CT scanning is increased [6].

While low and medium ROS concentrations are necessary to preserve basic physiological processes of the organism (e.g., regulation of NO^•^ synthesis, nicotinamide adenine dinucleotide phosphate oxidase NAD(P)H oxidase, vasoconstriction, cell adhesion), their high concentrations may affect excessive proliferation and cell apoptosis [7,8], resulting in further organ damage and functional deterioration of the system.

Specialized enzymatic antioxidant systems as well as numerous non-enzymatic systems preventing too high free radicals’ concentrations are components of all eukaryotic organisms’ cells. Basic components of antioxidant enzymatic system include catalase (CAT), superoxide dismutase (SOD) and enzymes involved in glutathione metabolism such as glutathione peroxidase (GPx) or glutathione reductase (GR), all interacting with each other. Thioredoxin reductase (TrxR) and other enzymes also play an antioxidative role.

On the other side, non-enzymatic antioxidants include endogenous compounds synthesized in the organism as well as natural and synthetic exogenous compounds. Some of them were until recently regarded only as inactive end products of metabolism, however, intensive studies of recent years confirm their antioxidant capacity, shedding new light on their biological role (including glutathione (GSH), bilirubin, albumin, ceruloplasmin or ferritin) [9,10].

The joint action of different antioxidants results in a greater protective effect than the summarized antioxidant effects of each compound separately. Literature states various definitions of total antioxidant capacity, including FRAP (ferric reducing ability of plasma) and FRASC (ferric reducing antioxidant activity and ascorbic acid concentration). Estimation of the total antioxidant capacity as a clinical exponent of oxidative stress is based on the determination of various markers’ concentrations in plasma, serum, saliva and other biological materials, e.g., single or total low molecular weight antioxidants, activity of the selected enzymes or markers of oxidative stress such as malonyldialdehyde (MDA) or phosphorylated histone H2 (γ-H2AX) as markers of lipid peroxidation or double-strand DNA breaks [11,12].

Methods for determining of total antioxidant capacity of plasma are based on the measurement of the ability of plasma to counteract the effects caused by ROS. Methods of measuring the total antioxidant capacity most commonly used in the literature are FRAP method which is a total ability of plasma to reduce Fe^3+^ ions; as well as its modification, FRASC, usually performed according to the Benzie and Strain method [13].

The total antioxidant capacity of plasma (FRAP/FRASC) is a result of the action of low molecular weight antioxidants (such as α-tocopherol, ascorbic acid, β-carotene, glutathione, uric acid, bilirubin), proteins (ceruloplasmin, ferritin, albumin, transferrin) and enzyme systems. Oxidative-stress-induced FRAP and FRASC decrease is eliminated by the increase in antioxidant enzymes activity with activation of non-enzymatic mechanisms. A decrease in FRAP/FRASC is observed in a later phase of oxidative stress and is caused by the depletion of antioxidant defense mechanisms [14,15].

A significant increase in FRAP/FRASC is also observed after supplementation with vitamins C, E, A and may be used in monitoring and optimization of antioxidative therapy [16].

Oxidative stress, although impossible to eliminate, has survived in the course of evolution together with the elements of antioxidant protection of the organism. Even though it is difficult to monitor in vivo due to the short viability of free radicals, the determination of enzymes limiting its development is possible and easily achievable due to the development of analytical methods examining enzymatic activities in biological material.

The antioxidant system associated with the operation of enzymes or non-enzymatic elements therefore presents a developed survival strategy, because it allows organisms to maintain an oxidative-antioxidative balance and eliminate oxidative stress which leads to disruption of redox signaling in cells and can initiate molecular damage.

A small amount of literature on the effect of ionizing radiation on the antioxidant system in humans became the subject of our interest, and informed the decision to undertake research determining the role of the antioxidative system and its impact on the human body against the harmful effects of ionizing radiation in routinely performed computed tomography (CT).

## 2. Materials and Methods

### 2.1. The Study Group

The study involved 35 adult patients of the Clinical University Hospital in Krakow, subjected to routine high-resolution computed tomography (HRCT) examination of the chest. They were selected as consecutive individuals who applied for routine HRCT examination of the chest. The examinations of chest were performed using a 16-row helical CT Siemens Somatom Sensation 16 scanner (Siemens Healthcare GmbH, Erlangen, Germany) and HRCT chest protocol, including CareDose option for dose reduction, based on X-ray beam modulation depending on patient’s body size. No i.v. contrast agent was used. The parameters of the protocol were as follows: tube voltage 120 kV, tube current 169–253 mA automatically adjusted using CareDose option, rotation time 0.5 s, spiral pitch factor 1.25, configuration of detectors 16 × 0.75 mm, slice thickness and increment 1 mm, data collection diameter 500 mm, reconstruction diameter 350 mm, convolution kernel B60f, CT window width 1200 HU, level −600 HU. During every CT examination, the radiation dose used was estimated by the scanner software and presented in final report as CT dose index computed tomography dose index CTDI_vol_ and dose-length product (DLP) values. CTDI [mGy] is a standardized measure of radiation dose during CT examination allowing comparison between different scanners. CTDI_100_ [mGy] is a linear measure of dose distribution using a 100-mm standard pencil dose chamber, which does not take into consideration the variation of a human body size and composition, thus having a very limited clinical value. CTDI_w_ (weighted) [mGy] uses measurements acquired at central and peripheral positions in the head or body phantoms, thus it is closer to the human dose profile as compared to CTDI_100_.

CTDI_w_ = 1/3 CTDI_100_ (center) + 2/3 CTDI_100_ (periphery)

CTDI_vol_ [mGy] is calculated by dividing CTDI_w_ by pitch factor.

DLP [mGy × cm] estimates the overall dose output, using multiplication of CT dose index by scan length.

DLP = CTDI_vol_ × scan length

Pathological changes were excluded in all participants of the study. Demographic data of patients as well as administered doses of ionizing radiation during HRCT examination are given in Table 1.

Patients (age-matched adults) completed the questionnaire regarding personal data and general disorders before the study. Smokers or people with history of smoking have been excluded from the study. The exclusion criteria also included systemic diseases such as diabetes and chronic inflammation, alcohol abuse, use of antibiotics, antioxidants or anti-inflammatory drugs in the last seven months. The study did not require the administration of a contrast agent. The study report has been approved by the bioethics committee of the Jagiellonian University in Kraków (KBET/223/B/2011). The study was conducted in accordance with the ethical principles of the Helsinki Declaration of 2008. All subjects expressed a written informed consent before taking part in the study.

### 2.2. Collection and Preparation of the Material

**Figure 1:**
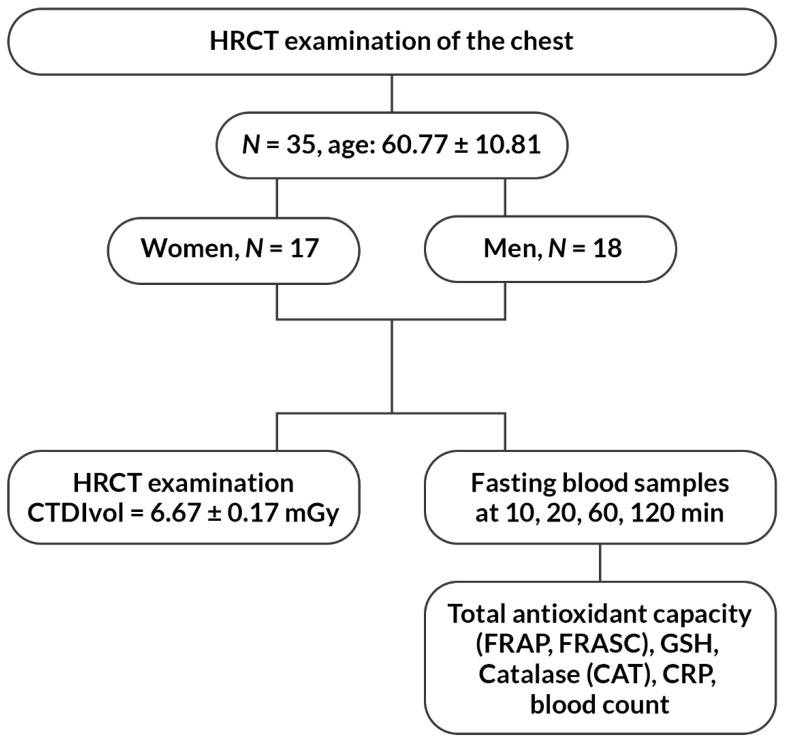
The flow chart of the study.

Fasting blood samples were collected from all 35 participants of the study in the morning directly prior to examination. The second blood collection took place 10 min after the examination, and other after 20, 60 and 120 min. In total, 10 mL of blood was collected from each patient from the ulnar vein puncture using a closed Sarstedt system (Sarstedt AG&Co., Numbrecht, Germany) containing K2EDTA as an anticoagulant. Plasma was separated from the blood cells by centrifugation at 400× *g* for 10 min at 4 °C. Plasma samples were stored at −80 °C until the analysis of the selected antioxidative markers: catalase (CAT), total antioxidant capacity of plasma expressed as FRAP and FRASC, and reduced glutathione (GSH). In addition, routine laboratory tests were performed from the obtained material (i.e., blood count and C-reactive protein (CRP) according to the applicable procedures in the hospital laboratory). The flow chart of the study is presented in Figure 1.

### 2.3. Analysis of Selected Antioxidant Parameters

CAT determination was performed using the Randox Laboratories Ltd. kit (London, UK) according to the manufacturer’s instructions.

FRAP determination was performed using the Benzie and Strain’s method based on using the ability of plasma to reduce Fe^3+^ ions (ferric reducing ability of plasma) [13].

In addition, FRACS (vitamin C) was designated for persons undergoing HRCT. The FRASC method is a simple modification of the FRAP method, which allows the simultaneous measurement of ascorbic acid and FRAP in the same sample [13,17]. FRASC was introduced with regard to the reference HPLC method for ascorbic acid [15].

The GSH assay was performed according to the Ellman’s method [18] as described in Darczuk et al. [19]. The method is based on the reaction of thiols with DTNB (5,5’-dithiobis-2-nitrobenzoic acid) chromogen, resulting in formation of a measured yellow 5-thio-2-nitrobenzoic acid dianion (TNB) [20].

The values of individual antioxidant parameters obtained in subsequent collection times were compared after 10, 20, 60 and 120 min separately in men and women.

### 2.4. Statistical Analysis

Analytic results have been evaluated using R 3.4.2 statistical package (R Foundation for Statistical Computing, Vienna, Austria). The differences between values of variables in two groups were tested by Mann-Whitney U test (nonparametric). Correlation between qualitative features was assessed with Spearman’s rank correlation coefficient (nonparametric). Multiple correlation coefficient was used to judge the relationship between more than two qualitative features. The differences between values in two repeated measurements (before HRCT examination and after 10/20/60/120 min) were tested by Wilcoxon paired-samples test (nonparametric). Significance was set at 5% level (*p* = 0.05).

## 3. Results

The presented study has analyzed the differences in selected antioxidant parameters of the oxidative-antioxidative system (CAT, GSH, FRAP, FRASC) at various time points before and after HRCT examination of the chest.

**Figure 2:**
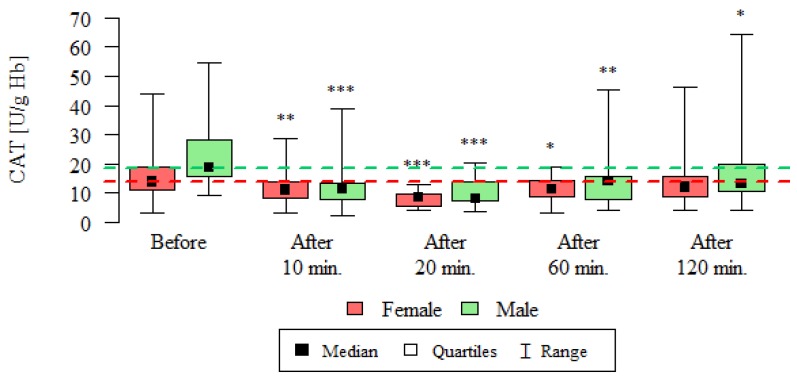
CAT activity [U/g Hb] in the blood of subjects undergoing routine HRCT examination before the test, 10 min, 20 min, 60 min and 120 min after the test. The analysis was carried out using the Wilcoxon test for dependent (repeated) measurements; the graph shows the medians, quartiles and ranges of values of individual variables. * *p*—comparison with the measurement before the examination (* *p* < 0.05, ** *p* < 0.01, *** *p* < 0.001)—Wilcoxon test for dependent (repeated) measurements.

**Figure 3:**
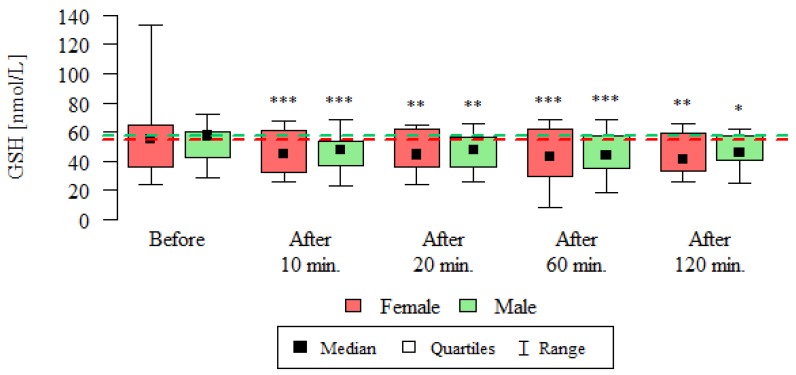
GSH concentration [nmol/L] in the plasma of subjects undergoing routine HRCT examination before the test, 10 min, 20 min, 60 min and 120 min after the test. The analysis was carried out using the Wilcoxon test for dependent (repeated) measurements; the graph shows the medians, quartiles and ranges of values of individual variables. * *p*—comparison with the measurement before the examination (* *p* < 0.05, ** *p* < 0.01, *** *p* < 0.001)—Wilcoxon test for dependent (repeated) measurements.

Catalase activity (CAT) and reduced glutathione concentration (GSH) are summarized in Table 2, Figure 2 and Figure 3.

The presented results of catalase activity are statistically significantly lower at all time-points compared to the activity before the test, except for the measurement after 120 min in women (Table 2, Figure 2).

The concentration of reduced glutathione was statistically significantly lower at all time-point compared to its pre-test concentration, both in women and in men (Table 2, Figure 3).

**Figure 4:**
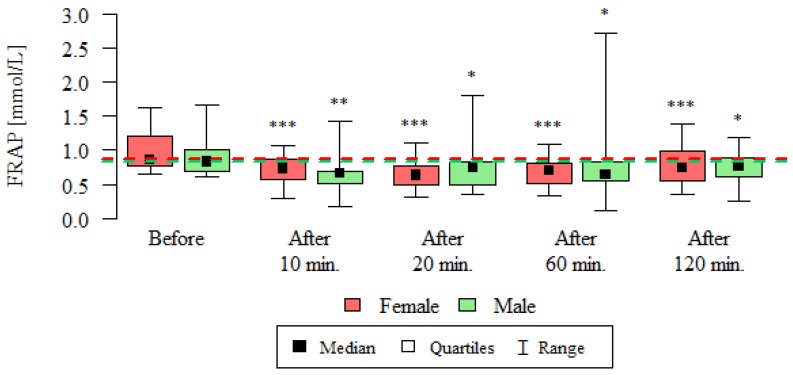
Total antioxidant capacity expressed as FRAP [mmol/L] in the blood of subjects undergoing routine HRCT examination before the test, 10 min, 20 min, 60 min and 120 min after the test. The analysis was carried out using the Wilcoxon test for dependent (repeated) measurements; the graph shows the medians, quartiles and ranges of values of individual variables. * *p*—comparison with the measurement before the examination (* *p* < 0.05, ** *p* < 0.01, *** *p* < 0.001)—Wilcoxon test for dependent (repeated) measurements.

**Figure 5:**
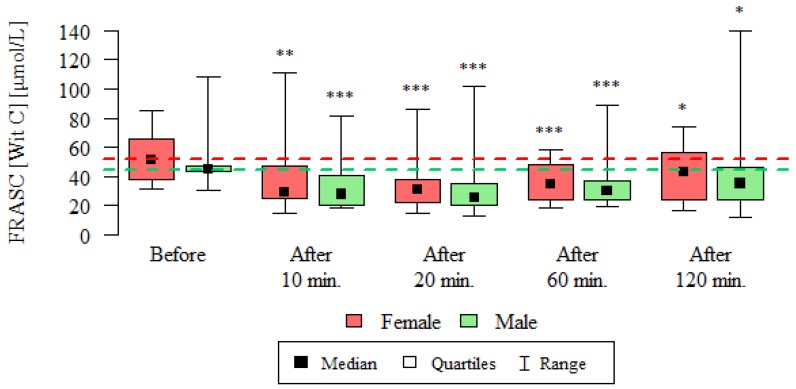
Total antioxidant capacity expressed as FRASC (vitamin C) [µmol/L] in the blood of subjects undergoing routine HRCT examination before the test, 10 min, 20 min, 60 min and 120 min after the test. The analysis was carried out using the Wilcoxon test for dependent (repeated) measurements; the graph shows the medians, quartiles and ranges of values of individual variables. * *p*—comparison with the measurement before the examination (* *p* < 0.05, ** *p* < 0.01, *** *p* < 0.001)—Wilcoxon test for dependent (repeated) measurements.

Consequently, the total plasma antioxidant capacity expressed as FRAP and FRASC was determined at before and after 10, 20, 60 and 120 min after the test. The results of these measurements are presented in Table 3, Figure 4 and Figure 5.

As the presented results show, the total antioxidant potential expressed as FRAP was statistically significantly lower after 10, 20, 60 and 120 min compared to its values before the test, both in women and men (Table 3, Figure 4).

Similarly, in the case of the antioxidative potential expressed as FRASC, its mean concentrations were lower at all time-point compared to its values before the test, both in women and in men (Table 3, Figure 5).

Subsequently, the relationship between the determined FRAP and FRASC parameters have been examined and summarized in Table 4.

The correlation FRAP and FRASC values after 10 min from the study was significant in the group of women, with a positive dependence. In the group of men, no relationship was observed between FRAP and FRASC at each of the time points (Table 4).

Subsequently, the influence of sex on the selected values of antioxidant parameters (CAT, GSH, FRAP and FRASC) have been examined and presented in Table 5.

Comparing the examined antioxidant parameters (CAT, GSH, FRAP and FRASC) depending on sex, it was noted that their mean values did not differ in the group of men and women in any of the time points.

The study also examined the influence of age on these antioxidant parameters separately for women and men and for the whole group. The results are shown in Table 6.

Comparing the examined parameters (CAT, GSH, FRAP and FRASC) depending on the age, it only affected the FRASC value significantly before the study, and the dependence was positive. In other cases of antioxidant parameters, age did not significantly affect their value, as presented in Table 6.

Subsequently, the influence of the radiation dose size (CTDI_vol_ and DLP) on the antioxidant parameters have been evaluated, with the results summarized in Table 7.

It was noted that CTDI_vol_ does not significantly affect the value of any antioxidant parameter. In turn, DLP significantly influences the average FRASC and GSH concentrations after 10 min from the study. The dependence on FRASC is negative, while the dependence on the reduced glutathione level is positive (Table 7).

## 4. Discussion

The effect of ionizing radiation on cells has been known since 1954 and relies heavily on free radical formation and reactions, which became the basis of the free radical theory of organisms’ aging.

The study examined the relationship between a single exposure to ionizing radiation during the CT examination of the chest and an antioxidant potential of blood expressed as the activity of selected antioxidant enzymes, i.e., catalase (CAT), reduced glutathione (GSH) and total antioxidant capacity (FRAP and FRASC) in blood plasma.

There are no studies in available, published scientific papers describing the participation of human antioxidative system in protection against harmful effects of ionizing radiation during routine imaging examinations. The presented works only address the topic of the influence of ionizing radiation on antioxidant systems in in vitro cell culture animal models.

Our study seems to be the first to try to explain the role of human antioxidant systems and their relationship with protection against the harmful effects of ionizing radiation.

The conducted study provides evidence that IR even in small doses causes the weakening of elements of the antioxidant system in humans during routine HRCT examination (doses in the 6.67 mGy range). The authors try to at least partially illuminate the scale of the problem through the analysis of the current state of knowledge on the impact of ionizing radiation on antioxidant systems in cell and animal models.

Liu et al. have studied the effect of ionizing radiation on the induction of oxidative stress in bone marrow mesenchymal stem cells (BMSCs) [21]. As a result of IR (6 Gy), there was an occurrence of intracellular increase of reactive oxygen species level (ROS), apoptosis and impaired ability of cell differentiation, although the mechanisms of observed changes have not been clarified. It was also noted that 6-Gy-level IR significantly increased the level of apoptotic cells (15.3 ± 2.67%) and ROS generation (a number of ROS-positive cells and mean fluorescence intensity were significantly higher than in 13 controls) compared to cells not exposed to IR (percentage of apoptotic cells equal to 5.73 ± 1.19%). Under the influence of IR, an increased expression/upregulation of reactive oxygen species-generating NADPH oxidase-4 (NOX4) was observed with simultaneous downregulation of SOD2. NOX4 belongs to NADPH-dependent oxidase family (NOX/DUOX) and physiologically participates in the production of ROS in various cell types. Superoxide-2-dismutase (SOD2) belongs to an iron-manganese superoxide dismutase family, providing cells with a capture of intracellular ROS levels. In addition, SOD2 plays an important role in inhibiting cellular apoptosis induced by an increase of ROS, IR and inflammatory cytokines [22]. It was found through a whole-body irradiation of mice that ROS generation was closely correlated with NOX4 upregulation and SOD2 downregulation in bone marrow damage [23]. The variable expression of NOX4 and SOD2 together with ROS level increase was closely correlated with an exposure to IR.

During our research, there was a similar visible failure of the antioxidant system elements, i.e., the decrease in catalase activity after 10, 20, 60 and 120 min after exposure to IR compared to its values before the exposure. The observed effect of a reduced regulation of antioxidant systems (CAT, GSH, FRAP, FRASC) after the exposure to IR is in accordance with the results of Liu et al., who associated a failure of the antioxidant system with microRNA upregulation (miR-22) [21].

MicroRNA (miRNA) belong to non-coding RNA genes that perform many functions, ranging from growth, differentiation and proliferation, to the regulation of total intracellular or mitochondrial reactive oxygen species production. It has been found that miRNA expression after irradiation (2 Gy) increases. It is not clear whether the radiation-induced increased expression of miR-22 plays a role in the regulation of IR-induced ROS production and cellular apoptosis, and subsequently in the impairment of bone formation.

Available literature shows the effect of miRNA on increased ROS production and the development of oxidative stress due to an increase of superoxidases levels and inhibition of antioxidant enzymes. The proposed mechanism of IR-induced miR-22 upregulation is associated with TGF-β increase and inhibition of SOD2 gene expression leading to a decrease in SOD2 activity and damage to irradiated tissues constituting a target for hydrogen peroxide in the failure of subsequent elements of the antioxidant system (mainly H_2_O_2_-degrading catalase) [24,25].

Han et al. have observed a decreased human umbilical vein endothelial cell (HUVEC) line viability, increased cytotoxicity and decreased migration ability in relation to the state before IR exposure (at 20 Gy dose) [26]. In addition, it has been shown that ionizing radiation increases ROS production, lipid peroxidation and oxidative DNA damage (through an increase of 8-OH-dG level) and reduces the activity of antioxidative enzymes (superoxide dismutase SOD, catalase CAT, glutathione S-transferase (GST) and glutathione peroxidase GPx) in HUVEC cells.

The IR exposure studies draw attention to the importance of multi-parameter analysis (multigene analysis or assessment of numerous biochemical markers), which presents different gene expression profiles or different behavior of biochemical transformation markers in whole blood samples irradiated with different X-ray doses.

Determination of IR limit doses in human imaging tests seems to be quite problematic, because such activities should not burden the patient. The estimated doses are effective based on calculations using phantoms as population-risk-associated IR doses. Research is being carried out on cell lines or animal models without actual assessment of IR damage to the human body. Hence, the identification of potential human biomarkers of IR exposure taking into account anatomical differences, different types of CT devices equipped with dose reduction options or using different types of CT protocols, seems to be so important.

El-Saghire et al. observed an increase the level of genes related to inflammation and immunity, increased secretion of growth factors, chemokines and cytokines after the exposure to 0.05 Gy IR, which indicates the activation of the immune response to such a dose of IR radiation [27]. On the other hand, IR application in a dose of 1 Gy, resulted in mobilization of cellular self-destructive pathways associated with the increase in mitochondrial ROS level, activation of p53-dependent apoptosis pathway, and consequently, DNA damage [27].

Vandevoorde et al. and Rothkamm et al. drew attention to the formation of double-stranded DNA breaks in the blood of adult patients through an increased level of phosphorylated H2 histone (γ-H2AX; 0.72 foci/cell) in response to X-ray CT compared to γ-H2AX level before IR exposure (0.56 foci/cell) [28,29].

Stephan et al. showed chromosomal abnormalities associated with a statistically significant increase in the level of dicentric and acentric chromosomal fragments under the influence of ionizing radiation at the dose of 12.9 mGy in pilot studies with blood samples from ten pediatric patients undergoing CT [30].

The increase in the number of DNA breaks and chromosomal aberrations in the form of dicentric chromosomes or deletions in lymphocytes and in whole blood under the influence of gamma radiation was also demonstrated in the work of Sudprasert et al. [31].

The vast majority of publications assessing the oxidative stress level associated with exposure to IR uses a double-strand DNA breaks testing (by assessing the level of phosphorylated H2 histone, γ-H2AX) in the blood of patients undergoing CT [32,33,34]. Double-strand DNA breaks (DSB) differ from single-strand DNA breaks as double lesions are more difficult to repair compared to single-strand ones (SSB). DSB more often lead to mutagenesis, cell death or neoplastic transformation than SSB [35].

Tissue sensitivity to radiation is different and depends to a large extent on the expression of genes responsible for apoptosis and damaged DNA repair.

In the Durante and Formenti’s study, IR-induced DNA damage was examined by assessing H2AX histone phosphorylation at Ser 15 (γ-H2AX) [35]. An increase in γ-H2AX level was observed in the cells of both irradiated lines, however, H460 cell line (with a lower SirT1 expression) is more sensitive to radiation compared to the A549 line. It proves that SirT1 expression is negatively correlated with radio-sensitivity. Activation of SirT1 by, e.g., resveratrol reduced the degree of DNA damage and cells apoptosis induced by radiation, however its blocking increased the damage degree (similar effect in both lines). While searching for the mechanisms of these reactions, it has been proven that SirT1 regulates apoptosis and radio-sensitivity of cells through the Sirt1/NF-κB/Smac pathway that may be a potential target in the treatment of non-small-cell lung carcinoma.

Available literature data confirm the hypothesis that the strategy of reducing radiation dose in CT in humans is associated with a parallel decrease in the γ-H2AX level constituting a type of biomarker of the exposure effect [36]. A wide range of activities have been undertaken globally to improve the effective and optimal CT dose, which is especially important in children, taking into account not only the need of reducing the dose, but also high resolution of diagnostic imaging. A number of studies reveal that even the same CT doses show statistically significant differences between study protocols for the same studies, suggesting that not all of the protocols have been properly standardized and optimized [37,38,39,40].

CT dose minimization activities are constantly evolving depending on imaging techniques as well as dose selection methods, which is of great importance in vulnerable groups such as children.

In the Mancuso et al.’s study, heterozygous mice had an increase in DNA oxidative damage (increase in γ-H2AX level), increased apoptosis and tumor induction after partial irradiation of the cerebellar tumor (3 Gy X-ray), which significantly stimulated hyperplasia thus promoting tumor growth [41]. A lower level of DNA damage (γ-H2AX <10–20 DSB) was observed in case of low ionizing radiation doses (<0.04 Gy), which did not cause changes in the cell cycle in human fibroblasts (no transition of G2 to M). At low IR doses, apoptotic cell death was found to be protective due to the selective elimination of damaged cells that might have contributed to the increase in tumorigenesis. In conclusion, the presented in vivo model using variable IR doses showed a clear carcinogenic potential for high doses of IR.

In addition to the dose of radiation, the age of the subjects plays a significant role in assessing the effects of IR exposure. Epidemiological data indicate that children have a higher relative risk of cancer than adults after using even low doses of radiation. Moreover, children, unlike adults, live longer after using cancer radiotherapy, which determines the need of setting clear and precise limits for the use of selected IR doses depending on the age. Published data in this area are modest, so it seems necessary to conduct studies demonstrating the X-ray-induced exposure effects in various age groups in order to select appropriate biomarkers of exposure and establish additional measures of radiological protection.

Lower doses and adequately lower risk reflect the technological progress that has been made in imaging examination using latest CT equipment in recent years. The results of the studies for smaller doses most often used in diagnostic radiology or epidemiological studies, are limited to in vitro and animal models, and in the case of humans are often unreliable because of too small sample size. This imposes the necessity of developing new algorithms for assessing IR exposure through the use of a number of biomarkers predictive about the harmfulness of the used IR dose.

The assessment of IR-induced biological damage through sole assessment of DSB DNA is mainly related to lymphocytes, which is a reflection of oxidative damage exclusively within one tissue–blood. However, one can assume that DNA damage combined with its repair in lymphocytes is representative of other physiological tissues. The assessment of only γ-H2AX level in X-ray exposure studies is biologically important but does not provide any insight into compensatory mechanisms regarding other macromolecules such as proteins or lipids that constitute the entire functioning of the antioxidant system.

## 5. Conclusions

1. A disorder of the antioxidative system was observed in patients after the exposure to ionizing radiation during HRCT examination of the chest.
2. The average activity of catalase as an element of human antioxidant defense was reduced after 10, 20, 60 and 120 min from the HRCT examination of the chest.
3. Mean glutathione concentrations and total antioxidant potential decreased after exposure to ionizing radiation in both women and men.
4. The observed changes in antioxidant parameters in humans are a manifestation of the oxidative-antioxidative balance disorder caused by the exposition to ionizing radiation during the routine HRCT examination of the chest.
5. Measurement of the selected antioxidant defense parameters may constitute a useful indicator of oxidative stress resulting from ionizing radiation.

## Figures and Tables

**Table 1:** Demographic characteristics of studied groups.

	Women	Men	Total
Number of patients	18	17	35
Age (mean ± SD)	61.61 ± 12.47	59.88 ± 9.04	60.77 ± 10.81
CTDI_vol_ (mean ± SD)	6.67 ± 0.17	6.67 ± 0.18	6.67 ± 0.17
DLP (mean ± SD)	227.89 ± 17.54	246.24 ± 22.64	236.8 ± 21.94

All values given as mean ± SD. Computed tomography dose index CTDI_vol_ which is a standardized measure of the radiation output of a CT system, measured in a cylindrical acrylic phantom; dose-length product DLP, which is the product of CTDIvol and the irradiated scan length.

**Table 2:** CAT (catalase) activity [U/g Hb] in blood cells and GSH (glutathione) concentration [nmol/L] in plasma of subjects undergoing routine HRCT (high resolution computed tomography) before the examination, 10 min, 20 min, 60 min and 120 min after the examination.

**Catalase Activity [CAT U/g Hb]**										
**Time**	**Sex**	***n***	**Mean**	**SD**	**Median**	**Min**	**Max**	**Q1**	**Q3**	*p* *
Before	Female	18	16.04	8.77	13.82	3.2	43.78	11.22	18.88	---
	Male	17	22.16	11.3	18.51	9.08	54.59	15.47	28.14	---
After 10 min	Female	18	11.59	5.85	11	2.97	28.64	8.36	13.75	0.002
	Male	17	12.53	8.28	11.4	2.33	38.79	7.65	13.15	<0.001
After 20 min	Female	18	8.24	2.66	8.56	3.93	12.93	5.67	9.89	0.001
	Male	17	10.4	5.17	8.2	3.51	20.27	7.32	13.95	<0.001
After 60 min	Female	18	11.12	4.32	11.32	3.22	19	8.7	14.42	0.024
	Male	17	15.18	10.62	14.2	3.91	45.16	7.94	15.82	0.005
After 120 min	Female	18	14.89	11.39	12	4.17	46.28	8.91	15.88	0.212
	Male	17	18.69	14.59	13.31	4.11	64.29	10.51	19.8	0.025
**Reduced Glutathione GSH [nmol/L]**										
**Time**	**Sex**	***n***	**Mean**	**SD**	**Median**	**Min**	**Max**	**Q1**	**Q3**	*p* *
Before	Female	18	54.35	24.31	54.78	24.04	132.85	36.14	65.04	---
	Male	17	52.93	12.21	57.24	28.91	72.15	42.27	60.53	---
After 10 min	Female	18	46.41	14.73	44.81	25.72	67.88	32.04	61	<0.001
	Male	16	46.25	12.65	47.22	23.37	68.02	36.52	53.3	0.001
After 20 min	Female	18	46.48	14.27	44.31	24.37	64.72	36.03	62.25	0.004
	Male	17	47.04	12.26	47.56	26.14	65.29	36.3	56.84	0.003
After 60 min	Female	18	43.56	17.63	43.27	8.08	68.08	29.39	61.85	0.001
	Male	17	44.89	14.02	43.78	18.45	68.55	34.87	57.44	<0.001
After 120 min	Female	18	45.15	14.35	41.26	26.22	65.92	33.15	59.35	0.002
	Male	16	46.58	11.45	45.65	25.05	62.38	40.73	56.88	0.013

Notes: The analysis was carried out using the Wilcoxon test for dependent (repeated) measurements; the table shows the medians, quartiles and ranges of values of individual variables. * *p*—comparison with the measurement before the examination—Wilcoxon test for dependent (repeated) measurements.

**Table 3:** Total antioxidant capacity expressed as FRAP (ferric reducing ability of plasma) and FRASC (ferric reducing antioxidant activity and ascorbic acid concentration) in the blood of subjects undergoing routine HRCT examination before the test, 10 min, 20 min, 60 min and 120 min after the test.

**FRAP [mmol/L]**										
**Time**	**Sex**	***n***	**Mean**	**SD**	**Median**	**Min**	**Max**	**Q1**	**Q3**	*p* *
Before	Female	18	1	0.31	0.87	0.64	1.63	0.77	1.2	---
	Male	17	0.91	0.31	0.83	0.61	1.66	0.69	1	---
After 10 min	Female	18	0.7	0.22	0.73	0.3	1.08	0.57	0.87	<0.001
	Male	17	0.65	0.28	0.66	0.17	1.42	0.52	0.69	0.004
After 20 min	Female	18	0.65	0.22	0.63	0.31	1.1	0.49	0.77	<0.001
	Male	17	0.74	0.34	0.74	0.35	1.8	0.5	0.83	0.02
After 60 min	Female	18	0.67	0.21	0.7	0.34	1.08	0.51	0.81	<0.001
	Male	17	0.8	0.57	0.64	0.13	2.73	0.56	0.83	0.017
After 120 min	Female	18	0.79	0.28	0.74	0.35	1.38	0.55	0.99	0.001
	Male	16	0.75	0.26	0.77	0.25	1.18	0.61	0.89	0.011
**FRASC (Vitamin C) [µmol/L]**										
**Time**	**Sex**	***n***	**Mean**	**SD**	**Median**	**Min**	**Max**	**Q1**	**Q3**	*p* *
Before	Female	18	52.43	15.79	51.3	31.53	84.72	37.84	65.61	---
	Male	17	48.72	17.75	44.7	30.32	108.46	42.95	47.35	---
After 10 min	Female	18	37.14	22.9	28.89	14.28	110.6	25	47.39	0.002
	Male	17	33.38	17.04	27.65	18.19	81.32	20.66	40.78	0.001
After 20 min	Female	18	34.14	18.62	30.69	14.23	86.1	22.12	37.62	<0.001
	Male	17	31.82	21.79	25.56	13.19	101.68	19.86	35.36	<0.001
After 60 min	Female	18	36.29	12.9	34.85	18.02	57.81	23.84	48.38	<0.001
	Male	17	34.95	16.09	30.27	19.28	89.03	24.41	36.66	<0.001
After 120 min	Female	18	42.72	19.68	43.29	16.51	73.68	24.39	56.66	0.018
	Male	16	40.36	29.59	35.03	11.99	139.97	23.85	45.88	0.039

Notes: The analysis was carried out using the Wilcoxon test for dependent (repeated) measurements; the graph shows the medians, quartiles and ranges of values of individual variables. * *p*—comparison with the measurement before the examination—Wilcoxon test for dependent (repeated) measurements.

**Table 4:** Correlation between FRAP and FRASC values in patients undergoing routine HRCT before the test, 10, 20, 60 and 120 min after the test.

Measurement	Correlation Coefficient	*p*	Dependence	The Power of Dependence
**Correlation between FRAP and FRASC in Women**				
Before	0.112	0.656	---	---
After 10 min	0.62	0.007	positive	average
After 20 min	0.183	0.467	---	---
After 60 min	0.005	0.987	---	---
After 120 min	−0.165	0.512	---	---
**Correlation between FRAP and FRASC in Men**				
Before	0.302	0.239	---	---
After 10 min	−0.006	0.981	---	---
After 20 min	0.228	0.377	---	---
After 60 min	−0.056	0.831	---	---
After 120 min	−0.076	0.78	---	---
**Correlation between FRAP and FRASC both Women and Men**				
Before	0.314	0.066	---	---
After 10 min	0.338	0.047	positive	weak
After 20 min	0.183	0.291	---	---
After 60 min	−0.017	0.922	---	---
After 120 min	−0.01	0.953	---	---

The analysis was carried out using the Spearman correlation coefficient.

**Table 5:** The influence of sex on mean CAT, GSH, FRAP and FRASC values at different time points of the CT: before the test and 10, 20, 60 and 120 min after the examination.

**CAT [U/g Hb]**										
**Time**	**Sex**	***n***	**Mean**	**SD**	**Median**	**Min**	**Max**	**Q1**	**Q3**	*p* *
Before	Female	18	16.04	8.77	13.82	3.2	43.78	11.22	18.88	0.041
	Male	17	22.16	11.3	18.51	9.08	54.59	15.47	28.14	
After 10 min	Female	18	11.59	5.85	11	2.97	28.64	8.36	13.75	1
	Male	17	12.53	8.28	11.4	2.33	38.79	7.65	13.15	
After 20 min	Female	18	8.24	2.66	8.56	3.93	12.93	5.67	9.89	0.4
	Male	17	10.4	5.17	8.2	3.51	20.27	7.32	13.95	
After 60 min	Female	18	11.12	4.32	11.32	3.22	19	8.7	14.42	0.318
	Male	17	15.18	10.62	14.2	3.91	45.16	7.94	15.82	
After 120 min	Female	18	14.89	11.39	12	4.17	46.28	8.91	15.88	0.198
	Male	16	18.69	14.59	13.31	4.11	64.29	10.51	19.8	
**GSH [nmol/L]**										
**Time**	**Sex**	***n***	**Mean**	**SD**	**Median**	**Min**	**Max**	**Q1**	**Q3**	*p* *
Before	Female	18	54.35	24.31	54.78	24.04	132.85	36.14	65.04	0.804
	Male	17	52.93	12.21	57.24	28.91	72.15	42.27	60.53	
After 10 min	Female	18	46.41	14.73	44.81	25.72	67.88	32.04	61	0.986
	Male	17	46.25	12.65	47.22	23.37	68.02	36.52	53.3	
After 20 min	Female	18	46.48	14.27	44.31	24.37	64.72	36.03	62.25	0.934
	Male	17	47.04	12.26	47.56	26.14	65.29	36.3	56.84	
After 60 min	Female	18	43.56	17.63	43.27	8.08	68.08	29.39	61.85	0.961
	Male	17	44.89	14.02	43.78	18.45	68.55	34.87	57.44	
After 120 min	Female	18	45.15	14.35	41.26	26.22	65.92	33.15	59.35	0.796
	Male	16	46.58	11.45	45.65	25.05	62.38	40.73	56.88	
**FRAP [mmol/L]**										
**Time**	**Sex**	***n***	**Mean**	**SD**	**Median**	**Min**	**Max**	**Q1**	**Q3**	*p* *
Before	Female	18	1	0.31	0.87	0.64	1.63	0.77	1.2	0.338
	Male	17	0.91	0.31	0.83	0.61	1.66	0.69	1	
After 10 min	Female	18	0.7	0.22	0.73	0.3	1.08	0.57	0.87	0.428
	Male	17	0.65	0.28	0.66	0.17	1.42	0.52	0.69	
After 20 min	Female	18	0.65	0.22	0.63	0.31	1.1	0.49	0.77	0.463
	Male	17	0.74	0.34	0.74	0.35	1.8	0.5	0.83	
After 60 min	Female	18	0.67	0.21	0.7	0.34	1.08	0.51	0.81	0.807
	Male	17	0.8	0.57	0.64	0.13	2.73	0.56	0.83	
After 120 min	Female	18	0.79	0.28	0.74	0.35	1.38	0.55	0.99	0.932
	Male	16	0.75	0.26	0.77	0.25	1.18	0.61	0.89	
**FRASC (vitamin C) [µmol/L]**										
**Time**	**Sex**	**N**	**Mean**	**SD**	**Median**	**Min**	**Max**	**Q1**	**Q3**	*p* *
Before	Female	18	52.43	15.79	51.3	31.53	84.72	37.84	65.61	0.347
	Male	17	48.72	17.75	44.7	30.32	108.46	42.95	47.35	
After 10 min	Female	18	37.14	22.9	28.89	14.28	110.6	25	47.39	0.656
	Male	17	33.38	17.04	27.65	18.19	81.32	20.66	40.78	
After 20 min	Female	18	34.14	18.62	30.69	14.23	86.1	22.12	37.62	0.335
	Male	17	31.82	21.79	25.56	13.19	101.68	19.86	35.36	
After 60 min	Female	18	36.29	12.9	34.85	18.02	57.81	23.84	48.38	0.478
	Male	17	34.95	16.09	30.27	19.28	89.03	24.41	36.66	
After 120 min	Female	18	42.72	19.68	43.29	16.51	73.68	24.39	56.66	0.398
	Male	16	40.36	29.59	35.03	11.99	139.97	23.85	45.88	

The analysis was carried out using the Mann-Whitney test. * *p* < 0.05; non-parametric analysis.

**Table 6:** The effect of age on mean CAT, GSH, FRAP and FRASC values at different time points of the CT scan: before the test and 10, 20, 60 and 120 min after the test.

Parameter	Before	After 10 Min	After 20 Min	After 60 Min	After 120 Min
**Females**					
FRAP [mmol/L]	*r* = −0.052 *p* = 0.839	*r* = 0.300 *p* = 0.227	*r* = 0.226 *p* = 0.366	*r* = 0.055 *p* = 0.829	*r* = −0.217 *p* = 0.387
FRASC (vitamin C) [µmol/L]	*r* = 0.543 *p* = 0.02 *	*r* = 0.417 *p* = 0.085	*r* = 0.46 *p* = 0.055	*r* = 0.286 *p* = 0.249	*r* = 0.323 *p* = 0.191
GSH [nmol/L]	*r* = −0.181 *p* = 0.472	*r* = −0.237 *p* = 0.344	*r* = −0.013 *p* = 0.958	*r* = −0.031 *p* = 0.903	*r* = 0.139 *p* = 0.583
CAT [U/g Hb]	*r* = −0.131 *p* = 0.603	*r* = −0.402 *p* = 0.098	*r* = −0.101 *p* = 0.69	*r* = 0.133 *p* = 0.598	*r* = −0.04 *p* = 0.874
**Males**					
FRAP [mmol/L]	*r* = 0.181 *p* = 0.488	*r* = 0.085 *p* = 0.746	*r* = 0.036 *p* = 0.892	*r* = −0.043 *p* = 0.87	*r* = 0.227 *p* = 0.398
FRASC (vitamin C) [µmol/L]	*r* = −0.392 *p* = 0.12	*r* = −0.403 *p* = 0.109	*r* = −0.084 *p* = 0.75	*r* = −0.346 *p* = 0.173	*r* = −0.631 *p* = 0.009 **
GSH [nmol/L]	*r* = 0.151 *p* = 0.563	*r* = 0.06 *p* = 0.824	*r* = −0.057 *p* = 0.828	*r* = −0.071 *p* = 0.786	*r* = 0.133 *p* = 0.624
CAT [U/g Hb]	*r* = 0.186 *p* = 0.476	*r* = 0.134 *p* = 0.608	*r* = −0.237 *p* = 0.36	*r* = 0.065 *p* = 0.804	*r* = 0.035 *p* = 0.897
**Females and Males**					
FRAP [mmol/L]	*r* = 0.048 *p* = 0.782	*r* = 0.218 *p* = 0.208	*r* = 0.117 *p* = 0.505	*r* = 0.03 *p* = 0.865	*r* = 0.043 *p* = 0.809
FRASC (vitamin C) [µmol/L]	*r* = 0.165 *p* = 0.343	*r* = 0.062 *p* = 0.723	*r* = 0.185 *p* = 0.287	*r* = 0.043 *p* = 0.806	*r* = −0.035 *p* = 0.842
GSH [nmol/L]	*r* = −0.014 *p* = 0.935	*r* = −0.064 *p* = 0.72	*r* = −0.028 *p* = 0.875	*r* = −0.04 *p* = 0.818	*r* = 0.123 *p* = 0.487
CAT [U/g Hb]	*r* = −0.03 *p* = 0.865	*r* = −0.125 *p* = 0.473	*r* = −0.181 *p* = 0.297	*r* = 0.068 *p* = 0.699	*r* = −0.059 *p* = 0.742

The analysis was carried out using the Mann-Whitney test. ** *p*, abnormality of distribution (** *p* < 0.01, * *p* < 0.05); non-parametric analysis.

**Table 7:** The effect of CTDI_vol_ and DLP radiation doses on the values of selected antioxidant parameters (CAT, GSH, FRAP and FRASC) of patients undergoing routine HRCT examination: before the test, and 10, 20, 60 and 120 min after the test.

Parameter	Before	After 10 Min	After 20 Min	After 60 Min	After 120 Min
**Dependence on CTDI_vol_**					
FRAP [mmol/L]	*r* = 0.059 *p* = 0.735	*r* = 0.14 *p* = 0.421	*r* = 0.178 *p* = 0.306	*r* = 0.011 *p* = 0.951	*r* = 0.156 *p* = 0.38
FRASC (vitamin C) [µmol/L]	*r* = −0.144 *p* = 0.408	*r* = −0.288 *p* = 0.094	*r* = −0.088 *p* = 0.614	*r* = −0.19 *p* = 0.274	*r* = −0.021 *p* = 0.907
GSH [nmol/L]	*r* = 0.226 *p* = 0.192	*r* = 0.151 *p* = 0.395	*r* = 0.131 *p* = 0.453	*r* = 0.296 *p* = 0.084	*r* = 0.107 *p* = 0.549
CAT [U/g Hb]	*r* = 0.071 *p* = 0.684	*r* = −0.051 *p* = 0.773	*r* = −0.18 *p* = 0.302	*r* = −0.067 *p* = 0.701	*r* = −0.026 *p* = 0.885
**Dependence on DLP**					
FRAP [mmol/L]	*r* = 0.052 *p* = 0.766	*r* = −0.208 *p* = 0.229	*r* = −0.116 *p* = 0.506	*r* = −0.062 *p* = 0.723	*r* = 0.021 *p* = 0.906
FRASC (vitamin C) [µmol/L]	*r* = −0.298 *p* = 0.082	*r* = −0.43 *p* = 0.01	*r* = −0.195 *p* = 0.261	*r* = −0.235 *p* = 0.174	*r* = −0.326 *p* = 0.06
GSH [nmol/L]	*r* = 0.238 *p* = 0.168	*r* = 0.176 *p* = 0.32	*r* = 0.129 *p* = 0.46	*r* = 0.192 *p* = 0.27	*r* = 0.141 *p* = 0.428
CAT [U/g Hb]	*r* = 0.257 *p* = 0.136	*r* = −0.015 *p* = 0.934	*r* = −0.14 *p* = 0.424	*r* = −0.088 *p* = 0.617	*r* = 0.103 *p* = 0.562

The analysis was carried out using the Spearman correlation coefficient.
